# Multiscale Approaches for Confined Ring Polymer Solutions

**DOI:** 10.1021/acs.jpcb.1c01953

**Published:** 2021-05-03

**Authors:** Iurii Chubak, Christos N. Likos, Sergei A. Egorov

**Affiliations:** † Faculty of Physics, University of Vienna, Boltzmanngasse 5, A-1090 Vienna, Austria; ‡ Sorbonne Université CNRS, Physico-Chimie des Électrolytes et Nanosystèmes Interfaciaux, F-75005 Paris, France; § Department of Chemistry, University of Virginia, Charlottesville, Virginia 22901, United States

## Abstract

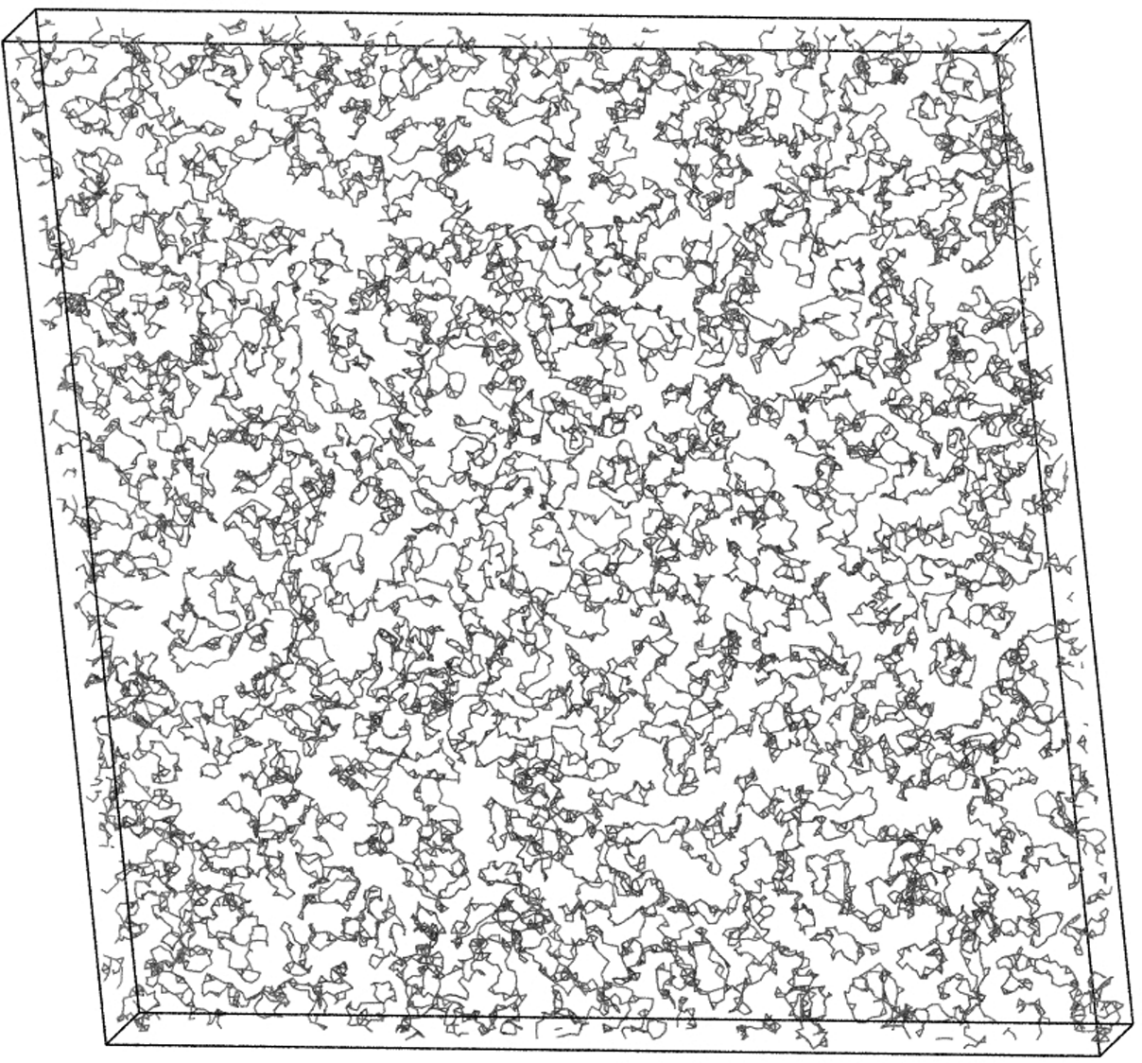

We apply a hierarchy of multiscale modeling approaches to investigate the structure of ring polymer solutions under planar confinement. In particular, we employ both monomer-resolved (MR-DFT) and a coarse-grained (CG-DFT) density functional theories for fully flexible ring polymers, with the former based on a flexible tangent hard-sphere model and the latter based on an effective soft-colloid representation, to elucidate the ring polymer organization within slits of variable width in different concentration regimes. The predicted monomer and polymer center-of-mass densities in confinement, as well as the surface tension at the solution-wall interface, are compared to explicit molecular dynamics (MD) simulations. The approaches yield quantitative (MR-DFT) or semiquantitative (CG-DFT) agreement with MD. In addition, we provide a systematic comparison between confined linear and ring polymer solutions. When compared to their linear counterparts, the rings are found to feature a higher propensity to structure in confinement that translates into a distinct shape of the depletion potentials between two walls immersed into a polymer solution. The depletion potentials that we extract from CG-DFT and MR-DFT are in semiquantitative agreement with each other. Overall, we find consistency among all approaches as regards the shapes, trends, and qualitative characteristics of density profiles and depletion potentials induced on hard walls by linear and cyclic polymers.

## 1 Introduction

Topological effects can have a profound impact on static and dynamic properties of polymers, being especially pronounced in melts and solutions of polymer rings. More specifically, the presence of topological nonconcatenation constraints in concentrated systems of rings enforces their compact, globular conformations that are very different from that of linear chains under similar conditions.^1−3^ Moreover, the lack of ends prohibits their relaxation via reptation and therefore significantly alters polymer dynamics.^4,5^ A consequence of the latter is a distinctive power-law stress relaxation in entangled ring polymer melts that does not feature a typical rubbery plateau in the case of the linear polymer melts.^4,6^ Another hallmark of ring polymer systems is the presence of the so-called threading constraints that correspond to the case when one ring pierces through the contour of another one. The impact of threading on the equilibrium dynamics of rings has not been entirely understood, as such constraints are usually hard to take into account in effective analytical models.^7,8^ Nevertheless, its effect has recently became amenable to analysis in computer simulations.^9−11^ Interestingly, a mutual threading of two rings slows down their diffusive relaxation^10,12^ and results in more correlated dynamics that have been conjectured to become glasslike for very long rings in concentrated solutions.^13^ While the required ring sizes for observing such phenomenon are not currently accessible either in experiments or simulations,^13^ a similar slow down of ring dynamics has recently been associated with enhanced threading in melts under nonequilibrium conditions.^14−16^

While the effects of ring topology are certainly more pronounced at higher system concentrations, they are found in dilute solutions as well.^17−23^ Notably, the nonconcatenation condition imposed on polymer loops without excluded volume alone leads to the Flory exponent of a self-avoiding random walk for the scaling of polymer size *R* with its polymerization degree *N*: *R* ∼ *N*^0.588^.^24,25^ Accordingly, more compact ring conformations in conjunction with topological constraints yield a very different form of the effective interaction potential between two coils *V*_eff_(*r*), which is usually defined in terms of the free energy penalty of placing two polymers at the center-of-mass separation *r*. In particular, in the case of rings one finds a distinctly non-Gaussian form of the effective potential that contrasts the Gaussian shape of *V*_eff_(*r*) for linear chains.^17,26−28^ More specifically, the effective interaction between two rings features a plateau at short center-of-mass separations *r* ≲ 0.5*R*_g,0_^ring^, where *R*_g,0_^ring^ is the ring’s radius of gyration at infinite dilution, with an amplitude of about 6–7 *k*_B_*T* for moderately sized rings with a polymerization degree *N* = 50–100.^26,27^ Interestingly, the ring conformations that contribute to the plateau region of *V*_eff_(*r*) at short center-of-mass separations are predominantly threading.^28^ For very long rings with *N* ≳ 2000, the amplitude of *V*_eff_(*r*) decreases to about 4.5 *k*_B_*T*; however, its shape otherwise remains unchanged.^26,28^

Many thermodynamics properties of ring polymer solutions in the dilute regime can be described using the effective representation of polymer coils as “soft colloids” interacting via effective potentials *V*_eff_(*r*).^29^ For instance, in our previous work,^19^ it was shown using a mean-field density functional theory (DFT) that the distinct form of *V*_eff_(*r*) for rings leads to a stronger tendency of rings to structure in planar confinement in comparison to the linear counterparts. Accordingly, the resulting form of the depletion potential *V*_dep_(*d*) between two walls immersed in a ring polymer liquid at a distance *d* is quite different from that in the case of linear chains at similar polymer concentrations, expressed in terms of the corresponding overlap values. In particular, the two cases differ both in terms of the shape of *V*_dep_(*d*) as well as in its value at *d* = 0, with the latter being deeper for rings. This highlights the rings as stronger depleting agents as compared to the liner chains. Furthermore, *V*_dep_(*d*) for rings^19^ somewhat resembles that of hard spheres by featuring a notable repulsive part with an oscillatory tail, both enhancing with increasing polymer concentration.

In this work, we consider in more detail the properties of ring polymer solutions confined between hard, repulsive wall using a series of multiscale modeling approaches. We employ a monomer-resolved DFT (MR-DFT), which we develop in section 2 for ring polymers of finite length *N* and molecular dynamics (MD) simulations, the details of which are given in section 3, as well as the mean-field DFT based on the soft-colloid representation summarized in section 4. The latter approach will be denoted as the coarse-grained DFT (CG-DFT) throughout this work. While CG-DFT is an approach based on eliminating degrees of freedom that can be important for large systems both in bulk and confinement, its accuracy may be very sensitive to the confinement strength and system density. Thus, a critical assessment of its validly is called for. Here, we provide such an assessment by systematically comparing CG-DFT to MD and MR-DFT. As presented here, MR-DFT is of particular interest, as it combines both the monomer degrees of freedom with flexibility of DFT approaches that are typically implemented in a grand canonical ensemble and feature readily available free energies. One of the major advantages of the latter is a direct access to thermodynamic quantities, such as surface tension, which are much more cumbersome to determine in MD. However, MD provides great flexibility in modeling interparticle interactions, while the MR-DFT is usually limited to hard-sphere potentials. So far, MR-DFT has been successfully used for both flexible and semiflexible linear polymer chains;^30−33^ however, its application for the ring architecture is rather scarce.^34^ The rest of the article is structured as follows. In section 5, we provide a monomer-resolved view on the problem at hand. In particular, we compare the monomer densities and surface tension at the interface as obtained from MD and MR-DFT. Additionally, we investigate the polymer organization within slits of variable width and the associated changes in the polymer conformation in contact with the hard walls at different densities. In section 6, we adopt a coarse-grained viewpoint. The results for the ring polymer organization in planar confinement from MD are compared to those from CG-DFT, with the latter showing quite good agreement with the former below the semidilute regime. Finally, we compare the polymer-induced depletion potentials as obtained from CG-DFT and MR-DFT, and we find good agreement between the two at comparable densities in the dilute regime, indicating rings as stronger depletants as compared to linear chains at similar volume fractions of the polymer component.

## 2 Monomer-Resolved Density Functional theory

### 2.1 Microscopic Model and Equation of State

In the monomer-based version of DFT used in this work, we model both linear and ring polymers using a freely jointed tangent hard-sphere model, which has been extensively used in our earlier DFT-based work on linear chains.^30−32^ In this model, each polymer consists of *N* tangent hard-sphere monomers of diameter σ; no bond-bending^33^ or torsional potentials are incorporated in this model so that the chains are fully flexible. For linear chains, all pairs of successive monomers (*i*, *i* + 1, with *i* = 1, ..., *N* – 1) are connected by a rigid bond of fixed length σ, with the monomers *i* = 1 and *i* = *N* corresponding to the two free ends. In the ring model used here, these two monomers are also connected by a rigid bond of length σ, so that all *N* monomers are completely equivalent.

We consider polymers under planar slit confinement between two hard walls. The monomer–wall interaction potential for the walls located at *z* = 0 and *z* = *d* has the form:
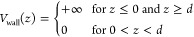
1

One of the key ingredients of the DFT discussed in section 2.2 is the equation of state (EOS) of the homogeneous polymer fluid. For the linear chains, we use the generalized Flory dimer (GFD) EOS obtained by Honnel and Hall.^35^ According to the GFD EOS, the compressibility factor *Z* for linear freely jointed tangent hard-sphere chains is given by^35^
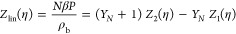
2where β = 1/*k*_B_*T*, *T* is the temperature, *P* is the pressure, and η = πρ_b_σ^3^/6 is the packing fraction of monomers with bulk density ρ_b_. In the present case, the bulk density ρ_b_ corresponds to a fixed chemical potential μ, which is the true variable in the grand canonical DFT.

In the above, the compressibility factor for the monomer hard-sphere fluid *Z*_1_(η) is taken from the Carnahan–Starling EOS:^36^
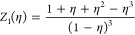
3while the compressibility factor for the tangent dimer hard-sphere fluid *Z*_2_(η) is taken from the Tildesley–Streett EOS:^37^
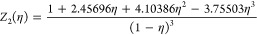
4Finally
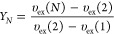
5where υ_ex_(*k*) is the volume excluded by a tangent hard-sphere *k*-mer to a hard-sphere monomer averaged over conformations of the *k*-mer. For *k* = 1, 2, and 3, the respective excluded volumes are^35^ υ_ex_(1) = 4πσ^3^/3, υ_ex_(2) = 9πσ^3^/4, and υ_ex_(3) ≈ 9.82605σ^3^, while for larger values of *k*, υ_ex_(*k*) can be estimated from^35^
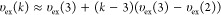
6

The above expression for the compressibility factor of linear chains can be written in the form:^38^
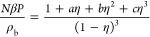
7where constants *a*, *b*, and *c* follow from eqs 2–6. Subtracting the ideal compressibility factor (*Z*_id_ ≡ 1) from eq 7 and using standard thermodynamic relations,^39^ one obtains the following result for the excess free energy per site of linear chains:^38^
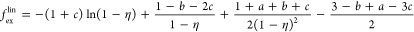
8which is one of the inputs required by DFT, as discussed in section 2.2 below.

For the ring polymers, we use the EOS obtained by Jiang et al:^34^
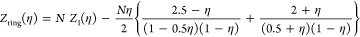
9which yields for the excess free energy per site of ring polymers:
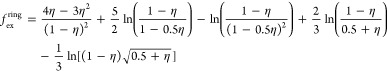
10

### 2.2 Density Functional Theory

The starting point of any DFT-based treatment^40,41^ is the expression of the grand free energy, Ω, as a functional of the polymer density profile ρ_p_(**R**_p_), where **R**_p_ = (**r**_1_, **r**_2_, ..., **r**_*N*_) is a collective variable with the individual monomer coordinates **r**_*i*_. The minimization of Ω with respect to ρ_p_(**R**_p_) yields the equilibrium polymer density distribution. The functional Ω is related to the Helmholtz free energy functional, *F*, via a Legendre transform:
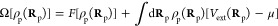
11where μ is the polymer chemical potential and *V*_ext_(**R**_p_) is the external field, which in the present case is due to the interaction of the polymer beads with the two walls:
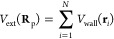
12

We employ the following approximation for the Helmholtz free energy functional, which separates it into ideal and excess parts according to^42^
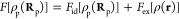
13with the ideal functional given by^42,43^
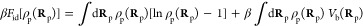
14while the excess term is a functional of the monomer density given by^42,43^
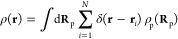
15

For the excess free energy functional, we adopt the weighted density approximation:^44^
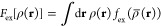
16with
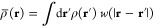
17In the above, *f*_ex_(ρ) is the excess free energy density per site of the polymer melt with site density ρ (which is obtained from the corresponding equation of state given in section 2.1), ρ̅(**r**) is the weighted density, and *w*(*r*) is the weighting function, which is normalized according to ∫d**r***w*(*r*) = 1. In the present work, we employ the simple square-well form for the weighting function, whose range is given by the diameter σ of the polymer segment:^45^
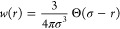
18where Θ(...) is the Heaviside step function. While more sophisticated forms of weight function are available in the literature (e.g., those used in the fundamental measure theory version of DFT),^46^ earlier studies^47^ have shown relative insensitivity of DFT results for polymeric systems to the specific choice of the weight function.

The minimization of the grand free energy functional Ω yields the following result for the equilibrium polymer density profile:
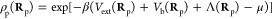
19where
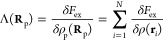
20

For our microscopic model, the binding energy for the linear chains has the form:
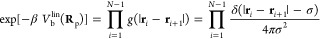
21while for the rings it takes the form:
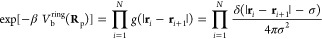
22with the convention **r**_1_ ≡ **r**_*N*+1_.

In order to obtain the segment density profile for linear chains, we now substitute the external field from eq 12, the excess free energy from eqs 8 and 16, and the bonding energy from eq 21 into eq 19. The resulting polymer density profile ρ_p_(**R**_p_) is then substituted into eq 15, which gives the following result for the segment density profile of linear chains:^42^
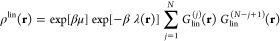
23where
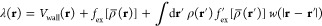
24
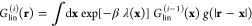
25and *G*^(1)^(**r**) = 1.

In order to obtain the segment density profile for ring chains, we substitute the external field from eq 12, the excess free energy from eqs 10 and 16 and the bonding energy from eq 22 into eq 19. The resulting polymer density profile ρ_p_(**R**_p_) is then substituted into eq 15 to give the following segment density for ring chains:
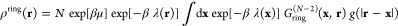
26with
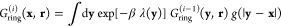
27and
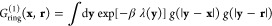
28where *N* ≥ 3, which is a necessary condition to form a ring.

In the limit *N* → ∞, the above DFT result for the ring segment density profile takes a simplified form:^48^
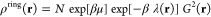
29where
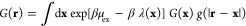
30with .

Due to its simplicity, the above result has been used (as an approximation) in an earlier DFT study^34^ of ring polymers of finite length *N*. To the best of our knowledge, no DFT implementation of eqs 26–28 for ring polymers of finite length *N* has been yet reported in the literature.

### 2.3 Numerical Implementation

For the planar confinement considered in this work, segment density profiles are functions of a single coordinate *z*. Thus, for linear chains, eq 23 takes the form^42^
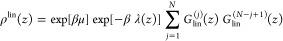
31with
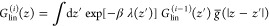
32and
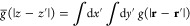
33

Likewise, for ring polymers under planar confinement, eq 26 takes the form
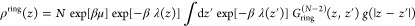
34with
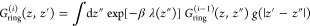
35and
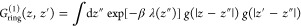
36

In our numerical implementation, we compute the density profiles on an equidistant grid along the *z* axis with the step *Δz* = 0.025σ. The eq 31 for the segment density profile of linear chains and the analogous equation for the ring polymers are both solved iteratively using Picard method, the tolerance criterion for terminating the iterative procedure is set to 10^–6^.

## 3 Molecular Dynamics Simulations

We performed molecular dynamics (MD) simulations of both ring and linear polymers chains confined within a slit of variable width *d*. Fully flexible polymer chains were modeled using the standard Kremer and Grest bead–spring model.^49^ The excluded volume potential between a bead pair was given by the Weeks–Chandler–Andersen (WCA) potential
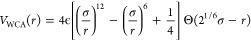
37where Θ(...) is the Heaviside step function. Since the potential (eq 37) is purely repulsive, it corresponds to polymer chains in good solvent conditions. Neighboring monomers along the polymer were connected via the finitely extensible nonlinear elastic (FENE) potential
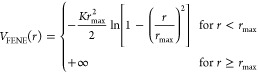
38with *r*_max_ = 1.5σ and *K* = 30ϵ/σ^2^. No bending was used in the present case.

In our simulations, steeply repulsive structureless walls were parallel to the *xy*-plane of a Cartesian coordinate system and always placed at *z* = 0 and *d*. The interaction between the wall and a monomer at the position **r** = (*x*, *y*, *z*) was similarly given by the WCA potential (eq 37):
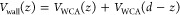
39where the first term corresponds to the wall at *z* = 0 and the second term to that at *z* = *d*. As usual, in the MD simulations σ was chosen as the unit of length, and the reduced temperature was set to unity: *k*_B_T* ≡ *k*_B_*T*/ϵ = 1. Finally, note that the MD model for polymer chains employed here differs from the one used in the MR-DFT, where the monomer–monomer and monomer–wall interaction potentials are completely hard. The reason is convenience in performing the MD simulations, but no effect is expected, since in the Kremer–Grest model the equilibrium bond length is stiff.

Ring and linear polymer solutions composed of chains of length *N* = 40, i.e., the same length as chains in the MR-DFT, were initialized in a large simulation box that was subsequently contracted to the final volume *V* = *L*_*x*_ × *L*_*y*_ × *d*. The values of *L*_*x*_ = *L*_*y*_ were chosen to match a given average monomer density ρ = *NM*/*V* in a slit of width *d*. In every case, we used *M* = 500 polymer chains. Typically, we considered monomer densities ranging from ρσ^3^ = 0.1 to ρσ^3^ = 0.6 with a step of 0.1. In addition, we highlight again that ρ of MD and ρ_b_ of MR-DFT generally do not coincide, since the former is an average, canonical density and the second is a proxy for μ; in other words, ρ_b_ is the monomer density of a bulk fluid that coexists with the confined one. To maintain the constant temperature *k*_B_*T* = ϵ, the system was coupled to a Langevin thermostat with γ = 0.1τ^–1^, where τ = σ(*m*/ϵ)^1/2^ and *m* is the monomer mass. The equations of motion of the system were integrated with the time step Δ*t* = 0.005τ. MD simulations were performed using the HOOMD-blue simulation package^50,51^ on graphics processing units (GPU’s). A typical MD run consisted of an equilibration phase with 10^7^ integration time steps and a subsequent up to 10^8^ steps long production phase. During a production run, monomer densities ρ(*z*) were measured on an equidistant grid along the *z*-axis with the step Δ*z* = 0.01σ, whereas polymer center-of-mass densities ρ_CM_(*z*) and polymer conformational properties (see Section 5) with the step Δ*z* = 0.1σ.

## 4 Coarse-Grained Density Functional theory

Similar to the MR-DFT, we employ a grand canonical CG-DFT, in which the grand potential Ω of the system is expressed through densities of the coarse-grained degrees of freedom.^39,40^ In our model, we will use effective pair interactions between two polymers’ centers of mass in combination with an effective interaction potential between a polymer’s center of mass and a hard wall. Therefore, Ω of CG-DFT is a functional of the center of mass polymer density ρ_CM_(**r**):
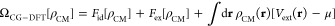
40where μ = constant is a fixed value of the fluid’s chemical potential, *V*_ext_(**r**) is the external potential, i.e., the effective potential between a polymer’s center of mass and a hard wall, *F*_id_[ρ_CM_] is the ideal contribution to the free energy
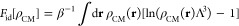
41with β = 1/*k*_B_*T* and  (*m*_p_ is the polymer’s mass), and the excess free energy *F*_ex_[ρ_CM_] is modeled here with the mean-field functional
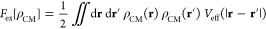
42with *V*_eff_(*r*) being the effective pair potential between two polymers’ centers of mass. As *V*_eff_(*r*) for both ring^17,27,28,52^ and linear^29,53^ polymers belongs to the class of ultrasoft pair potentials, i.e., fulfilling the condition ∫_0_^∞^ d*r r*^2^*V*_eff_(*r*) < ∞, it is expected that the mean-field theory (eq 42) will provide reasonably accurate results given that the polymer concentration is below the semidilute regime.^54−57^ We nevertheless extend our analysis to higher densities in order to explore in detail the limits of applicability of the models at hand.

The equilibrium center of mass density profiles are obtained by minimizing the grand potential (eq 40) with respect to ρ_CM_(**r**), yielding the following integral equation:
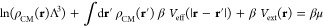
43where the fixed chemical potential μ can be expressed through the density of the polymer fluid in the bulk, ρ_bp_:
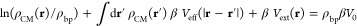
44with *V*_0_ = ∫ d**r***V*_eff_(|**r**|). Above, we used the fact that  for the bulk fluid’s free energy *F*(ρ_bp_) = *M*β^–1^ [ln(ρ_bp_Λ^3^) – 1] + ^1^/_2_(*M*ρ_bp_*V*_0_), and *M* denotes the number of polymer chains in the system. Finally, we note that the bulk polymer density ρ_bp_, which corresponds to some fixed chemical potential μ in the grand canonical ensemble, is not the same as the average polymer density ρ_p_ ≡ *M*/*V* that is typically used in MD in the canonical ensemble. To accurately compare the results from MD and CG-DFT, it is therefore necessary to relate ρ_p_ with ρ_bp_, and we will treat this issue systematically in the sections to follow.

In this work, we use the effective pair potential *V*_eff_(*r*) between the centers of mass of two ring polymers obtained in ref (27) and modeled by the following analytical expression:^17^
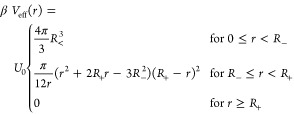
45with the parameters *U*_0_ = 1.434[*R*_g,0_^ring^]^−3^, *R*_>_ = 1.419*R*_g,0_^ring^, *R*_<_ = 1.000*R*_g,0_^ring^, and *R*_±_ = *R*_>_ ± *R*_<_. Here, *R*_g,0_^ring^ denotes the radius of gyration (see section 5 for an explicit definition) of a ring polymer at infinite dilution. The potential (eq 45) was derived for two isolated rings at infinite dilution, thereby limiting its applicability to dilute polymer concentrations. For the effective ring–wall interaction *V*_eff_^wall^(**r**) ≡ *V*_eff_^wall^(*z*) (assuming that the walls are orthogonal to the *z*-direction in the given system geometry), we utilize the potential obtained in ref (19) for rings of length *N* = 40 that can be accurately fit using *V*_eff_^wall^(*z*) = *a*_0_ exp(*a*_1_*z* + *a*_2_*z*^2^ + *a*_3_*z*^3^) with *a*_0_ = 64.1620(4), *a*_1_ = −8.360(30), *a*_2_ = 7.657(39), and *a*_3_ = −3.885(18). Whenever CG-DFT results are presented for linear chains, we use the Gaussian effective potential between the polymers’ center of mass developed in ref (58) in combination with the effective external potential for linear chains from ref (19).

Due to a series of different length scales used in this work, for the sake of clarity let us again review the quantities presented in the following sections as well as the units employed. In section 5, we mainly focus on the comparison between the results obtained with the MR-DFT and MD, thus the monomer diameter σ is adopted as the unit of length. We typically compare monomer density profiles *h*(*z*) = ρ(*z*)/ρ – 1, where ρ(*z*) is the monomer density within a slit and ρ = *NM*/*V* is the average monomer density. To highlight the differences between rings and linear chains, it is also interesting to consider polymer center-of-mass profiles *h*_CM_(*z*) = ρ_CM_(*z*)/ρ_p_ – 1, where ρ_CM_(*z*) is the polymer center-of-mass density in a slit and ρ_p_ = *M*/*V* is the average polymer density, respectively. The average polymer density ρ_p_ is related to the average monomer density ρ through ρ_p_ = ρ/*N* with *N* being the polymer length. In section 6, we focus on the comparison between CG-DFT with MD and the MR-DFT. As the infinite dilution radius of gyration *R*_g,0_ of polymers is the only relevant length scale in CG-DFT (eq 40), we adopt it as the unit of length throughout that section. For the two polymer architectures considered, we find *R*_g,0_^lin^ = 4.125σ and *R*_g,0_^ring^ = 3.064σ for chains of length *N* = 40 using the MD model outlined in section 3. In addition, it is convenient to use the express the average polymer densities ρ_p_ in terms of the polymer overlap density ρ_p_^*^ = 3/(4*πR*_g,0_^3^) that demarcates the onset of the semidilute regime. In terms of the monomer units, we have ρ_p_^*^σ^3^ = 0.0034 for linear chains and ρ_p_^*^σ^3^ = 0.0083 for rings.

## 5 Monomer-Resolved Viewpoint

### 5.1 Polymer Solutions in Contact with a Wall

**Figure 1:**
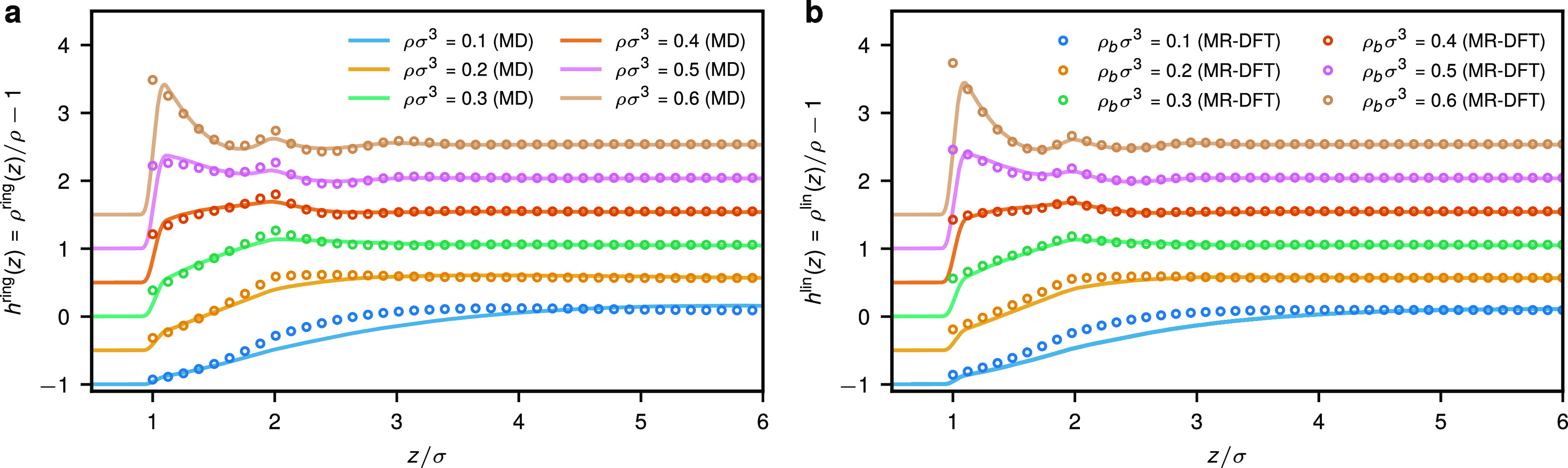
Monomer density profiles for (a) ring and (b) linear polymers confined within a broad slit, effectively resembling a contact with a single hard wall, from MD (solid lines) and monomer-resolved DFT (open circles). The MD results were obtained for average monomer densities ρ indicated in the legend of panel (a), whereas the MR-DFT results correspond to bulk densities ρ_b_ in a grand canonical ensemble indicated in the legend of panel (b). While we impose ρ_b_ = ρ_middle_ in MR-DFT, this does not necessarily hold true in MD. MD and DFT results were obtained from simulations of polymer solutions in a slit of width *d* = 50σ ≫*R*_g_^ring/lin^. For the sake of clarity, the profiles for consecutive densities have been shifted vertically by a value 0.5.

We begin by presenting the structure of ring and linear polymer solutions, as obtained from MD and MR-DFT, consisting of chains of length *N* = 40 confined within a broad slit of width *d* = 50σ. For this *N*, such an arrangement effectively corresponds to polymer solutions in contact with a single hard wall. To take into account the difference between monomer-wall interaction potentials in MD (eq 39) and MR-DFT (eq 1), the walls in MR-DFT were placed at *z* = σ and *d* – σ (that is, effectively *d*_MR–DFT_ ≈ *d*_MD_ – 2σ). Figure 1 shows the monomer density profiles *h*(*z*) = ρ(*z*)/ρ – 1 for the ring and linear polymer case at various densities from MD and MR-DFT. In the MR-DFT, the results correspond to bulk densities ρ_b_ in a grand canonical ensemble (taken to be the same as ρ) and a constraint that the density in the middle of the slit coincides with ρ_b_: ρ_middle_ ≡ ρ(*d*/2) = ρ_b_. In contrast, in MD the results were obtained from simulations at a fixed average monomer densities ρ in the *NVT* ensemble. Due to the latter, the enhanced depletion of polymer chains at the confining walls^59^ leads to ρ_middle_ that deviates from ρ. In particular, in MD we find ρ_middle_σ^3^ = 0.1096, 0.2124, 0.3143, 0.4160, 0.5176, and 0.6196 compared to ρ_middle_σ^3^ = 0.1, 0.2, 0.3, 0.4, 0.5, and 0.6 in MR-DFT, respectively. In summary, the difference in the simulation ensembles leads to a somewhat different average monomer average density ρ = *d*^–1^ ∫_0_^*d*^ d*z* ρ(*z*) in the slit in the MR-DFT that is about 3–10% different from ρ_b_. Accordingly, in Figure 1 we normalize the MR-DFT monomer density profiles with observed average density ρ. Despite such a discrepancy between the two modeling approaches, we find very good agreement between the final density profiles in MD and MR-DFT. For both rings and linear chains, MR-DFT reproduces the location of density peaks as well as main developments of the profiles (see Figure 1a,b) at both lower and higher concentrations (also note the difference in the employed MD and MR-DFT models). This indicates that MR-DFT is a robust approach that is expected to deliver accurate structure at concentration where more coarse DFT methods based on soft potentials are no longer applicable.

**Figure 2:**
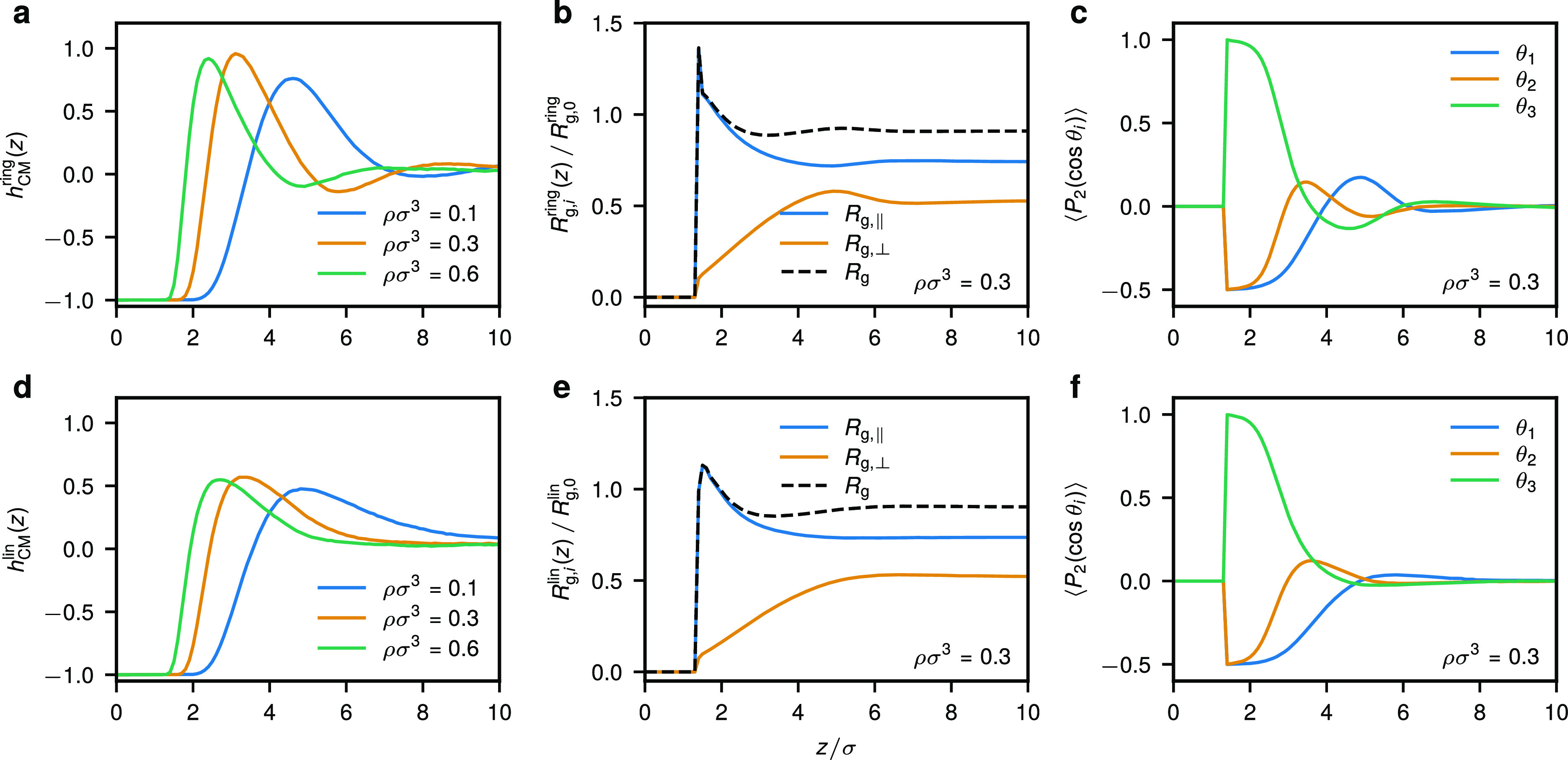
Organization of ring (top row) and linear (bottom row) polymer chains in contact with a hard repulsive wall. Center-of-mass density profiles for (a) ring and (d) linear polymers at different average monomer densities ρ. The total *R*_g_, parallel to the wall *R*_g,∥_, and orthogonal to the wall *R*_g,⊥_ radius of gyration of a polymer for (b) rings and (e) linear chains as a function of the distance *z* away from the wall for ρσ^3^ = 0.3. The alignment of the three eigenvalues of a polymer’s gyration tensor with the confining wall quantified by means of the second Legendre polynomial (eq 48) for (c) rings and (f) linear chains as a function of *z* for *ρσ*^3^ = 0.3. The results presented here correspond to polymer solutions confined in a broad slit of width *d* = 50σ. Qualitatively similar dependence of the conformational properties on *z* is obtained for other densities (see Figures S1 and S2). The results presented here were obtained in MD.

As shown in Figure 2, ring polymers feature a higher tendency to structure at the confining wall, as compared to the linear ones. We illustrate this using the MD polymer center-of-mass density profiles *h*_CM_(*z*) = ρ_CM_(*z*)/ρ_p_ – 1 (where ρ_p_ = *M*/*V*) is the average polymer concentration within the slit) for different monomer densities ρ, as seen in Figure 2a,d. We find that linear chains feature a single density peak followed by a uniform distribution of chains as indicated by a horizontal profile of *h*_CM_^lin^(*z*) (see Figure 2d). The rings similarly exhibit the first main peak, which is however more pronounced as compared to the linear counterparts, followed by a shallow dip in *h*_CM_^ring^(*z*) and another very small secondary peak (see Figure 2a and S1). Such spatial organization is preserved at all densities ρ considered for both architectures, although in both cases the main peaks are shifted toward the confining wall, demonstrating enhanced aggregation of polymers in the respective region. In summary, these results confirm our previous analysis based on a mean-field DFT that employed a soft particle representation of ring and linear polymers valid at dilute concentrations^19^ (a quantitative comparison is given in the section 6). For the average monomer density ρσ^3^ = 0.3, we have ρ_p_/ρ_p_^*^ ≈ 0.9 for ring, i.e., about the onset of the semidilute regime, and ρ_p_/ρ_p_^*^ ≈ 2.2 for linear polymers. Here, ρ_p_^*^ = 3/(4π*R*_g,0_^3^) is the overlap concentration of the respective species and *R*_g,0_ is the polymer’s radius of gyration at infinite dilution, as defined below.

To gain a better understanding of the fluid structure within the slit and its layering at the walls, we additionally quantify the size and orientation of polymer conformations in MD using the three eigenvalues λ_1_, λ_2_, and λ_3_ (λ_1_ ≥ λ_2_ ≥ λ_3_) and the associated normalized eigenvectors *ê*_1_, *ê*_2_, and *ê*_3_ of the radius of gyration tensor *G*, whose components are defined as follows:
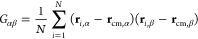
46where **r**_*i*_ is the position of the *i*th monomer,  is the center of mass position of the polymer, and α and β = 1, 2, and 3 denote the three Cartesian components. The polymer’s radius of gyration is then given by *R*_g_ ≡ ⟨*R̂*_g_^2^⟩^1/2^ with *R̂*_g_^2^ = λ_1_ + λ_2_ + λ_3_, where ⟨...⟩ denotes a statistical average over conformations and a hat stands for instantaneous values. Using the eigenvectors and corresponding eigenvalues of *G*, we quantify the average extension of a polymer chain in the directions parallel and perpendicular to the walls, *R*_∥_ ≡ ⟨*R̂*_∥_^2^⟩^1/2^ and *R*_⊥_ ≡ ⟨*R̂*_⊥_^2^⟩^1/2^, respectively, given by
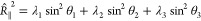
47a
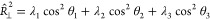
47bwhere θ_*i*_ is the angle between the eigenvector *ê*_*i*_ and the *z*-axis (perpendicular to the confining walls), implying that cos θ_*i*_ = *ê*_*i*_·*ẑ*. In addition, a random orientation of polymers in the slit results in *R*_⊥_^2^ = *R*_g_^2^/3 and *R*_∥_^2^ = 2*R*_g_^2^/3. Finally, the orientation of a polymer in the slit can be quantified by means of the orientation of the eigenvectors *ê*_*i*_ using the second Legendre polynomial
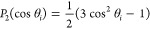
48whose average yields 0, if the eigenvector *ê*_*i*_ features no preferential orientation along *ẑ*. However, its average gives 1 or −0.5, indicating that *ê*_*i*_ is aligned with *ẑ* or lies orthogonally to it, respectively.

In the following section, we will focus on the conformational properties for polymer solution at ρσ^3^ = 0.3, although essentially the same trends apply to other densities as well (compare Figures 2 to S1 and S2). Naturally, the ring and linear polymer chains located in close proximity to the confining wall feature extended conformations that almost entirely lie in the *xy*-plane, as seen from vanishing values of *R*_g,⊥_ in Figures 2b,a. This is further supported by the fact that ⟨*P*_2_(cos θ_1_)⟩ and ⟨*P*_2_(cos θ_2_)⟩ approach −0.5 for the two larger eigenvalues of the polymer’s gyration tensor, whereas for the smallest one, we find ⟨*P*_2_(cos θ_3_)⟩ ≃1 at small wall separations. For both architectures, we find that at the position of the main density peak (*z* ≈ 3σ) the average polymer size is about the same as in the bulk, albeit being more extended in the in-plane direction. Furthermore, at this point we still find that the largest eigenvalue λ_1_ lies predominantly in the *xy*-plane, whereas the middle one λ_2_ becomes almost isotropic with a slight tendency to align with the *z*-axis (see Figure 2c,f). As we move away from the wall, in the case of linear chains the polymer’s orientation gradually becomes isotropic, and *R*_g,⊥_ and *R*_g,∥_ approach monotonously their bulk values (Figures 2e,f), flattening at about the point where the corresponding center-of-mass density profile becomes horizontal (Figure 2a). Interestingly, while such behavior is generally similar in the case of rings (Figures 2e,f), there exist certain differences pertinent to the layer of reduced density seen for polymer rings (4.5 ≲ *z*/σ ≲ 8 for ρσ^3^ = 0.3 in Figure 2a), namely, in the latter layer the rings feature maximal *R*_g,⊥_ and positive values of ⟨*P*_2_(cos θ_1_)⟩ combined with negative values of ⟨*P*_2_(cos θ_2_)⟩ and ⟨*P*_2_(cos θ_3_)⟩, indicating a tendency toward reorientation as compared to the first ring layer in the wall proximity. As we show below, such a structural arrangement of rings can even become amplified by confining polymer solutions to narrower slits, where the density correlations become stronger.

We now assess the effect of polymer architecture on the surface tension γ of the liquid–wall interface. In MD, we compute the surface tension using the Irving and Kirkwood approach^60−62^ based on evaluating the normal *P*_N_(*z*) and tangential *P*_T_(*z*) components of the pressure tensor within the slit and then integrating their difference:
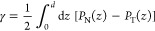
49where the equation above assumes that there are two hard walls in the system, located at *z* = 0 and *z* = *d*, respectively. Accordingly, the two components of the pressure at the position *z* can be evaluated as follows:^61,62^
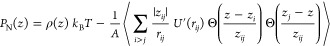
50
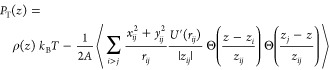
51where ρ(*z*) is the monomer density at *z*, *A* ≡ *L*_*x*_ × *L*_*y*_ is the interface area, **r**_*ij*_ = **r**_*j*_ – **r**_*i*_, *U*′(*r*_*ij*_) is the derivative of a pair potential, and Θ(...) is the Heaviside step function. The sum in eqs 50 and 51 runs over different particle pairs, and for any pair contribution between particles at **r**_*i*_ and **r**_*j*_, one needs to update the pressure components for all *z* that lie between *z*_*i*_ and *z*_*j*_. The contributions from the wall-monomer interactions can be similarly computed using eqs 50 and 51 by taking into account that the tangential part is identically zero. Finally, note that eqs 50 and 51 applies only in the case of pair interactions between particles, and other expressions are necessary in the case of three-body or multibody forces.^62^ The surface tension γ in the MR-DFT was computed from equilibrium monomer density profiles as detailed in ref (59).

**Figure 3:**
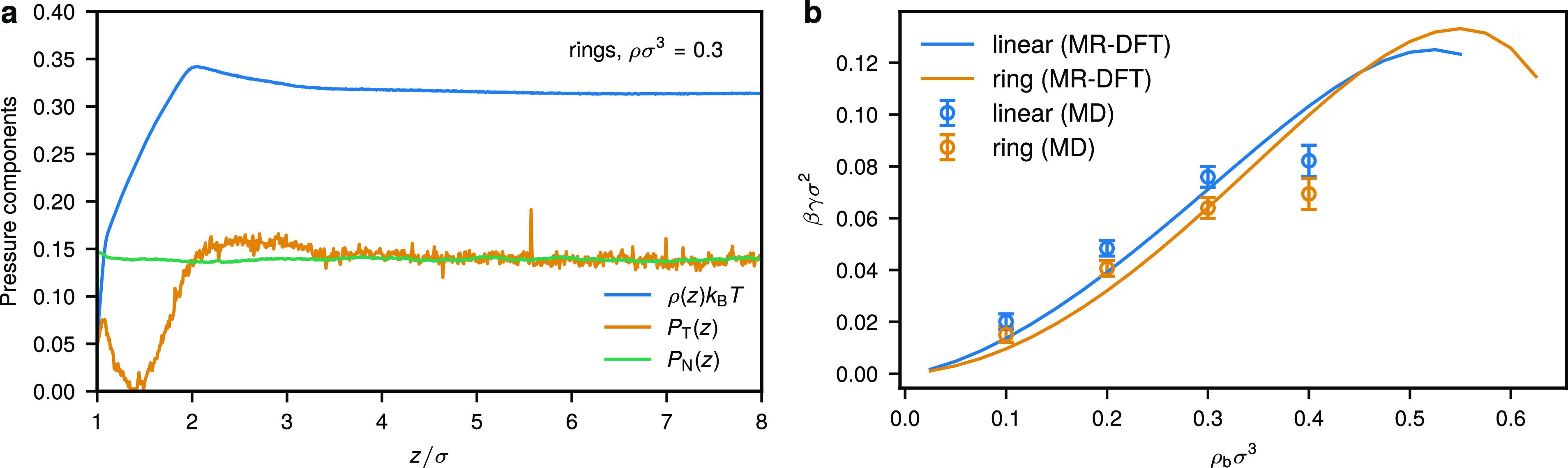
(a) Tangential *P*_T_(*z*) and normal *P*_N_(*z*) components of the pressure as a function of the distance away from the wall for a ring polymer solution at ρσ^3^ = 0.3. The profiles for linear chains are very similar. (b) Surface tension for ring and linear solutions in contact with a hard repulsive wall as extracted from MR-DFT for different monomer bulk densities ρ_b_ (solid lines) and MD at different average monomer densities in the slit ρ (open circles).

In Figure 3, we show the surface tension γ at the interface as obtained from MD and MR-DFT. Figure 3a shows the dependence of the normal *P*_N_(*z*) and tangential *P*_T_(*z*) pressure components at the interface for rings at ρσ^3^ = 0.3 (we find very similar profiles for linear chains at the same densities). As expected, due to mechanical stability,^61^ we find a constant profile for *P*_N_(*z*) ≡ constant, and variations in *P*_T_(*z*) with both components becoming equal further into the slit. In Figure 3b, we compare the surface tensions as obtained from MD at different average monomer densities ρ and the MR-DFT at different bulk monomer densities ρ_b_. To enable a comparison with the MR-DFT, we assumed that the interface in MD is effectively located at *z* ≈ σ; thus, the integral over the pressure component difference *P*_N_(*z*) – *P*_T_(*z*) in (eq 49) runs between *z*_1_ = σ and *z*_2_ = *d* – σ. We find very good agreement between the surface tensions in MD and MR-DFT at lower monomer densities (ρσ^3^ ≲ 0.3), but some differences are observed for ρσ^3^ ≳ 0.3, where the MR-DFT yields higher values of γ than in MD. Such a difference might stem from the approximations employed in the EOS, as even small deviations in the density profiles can lead to notable changes in the free energy that is used for computing the surface tension. From both methods, we systematically observe that rings feature a somewhat smaller γ than in the case of linear chains. Although it indicates that at the fixed monomer density linear chains generate a stronger attraction between the walls immersed into the polymer fluid (lim_*d*→0_*V*_dep_(*d*) = −2γ), the conclusion turns out to be the opposite if one compares ring and linear polymer solutions at the same polymer volume fraction, i.e., in terms of the polymer overlap concentration as discussed further in section 6.

### 5.2 Polymer Solutions Confined between Two Hard Walls

**Figure 4:**
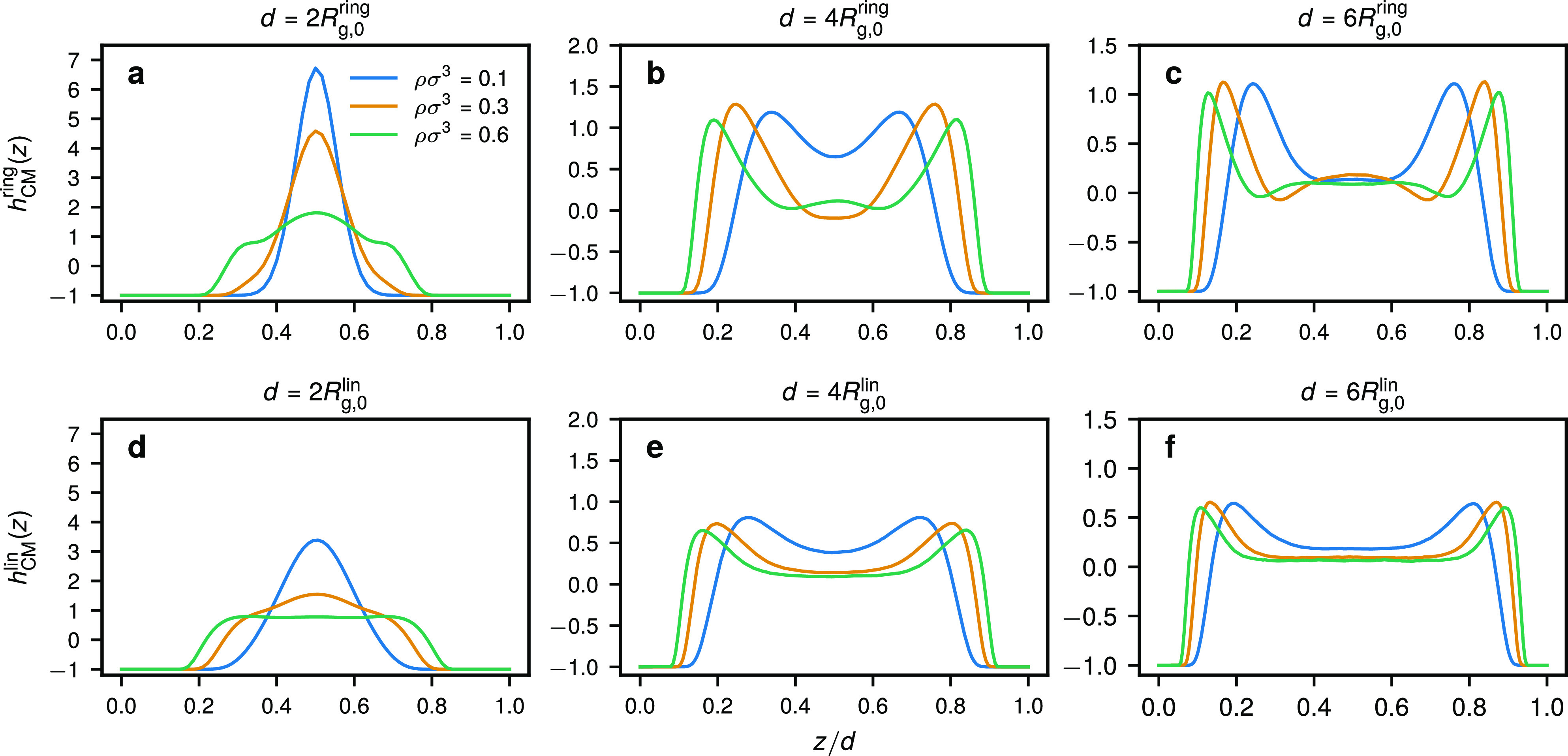
Center-of-mass density profiles for (a–c) ring and (d–f) linear polymers confined within a slit of width *d* for different values of *d* and average monomer densities ρ obtained from MD. The legends in panels (b–f) are the same as that in panel (a).

We now consider ring and linear polymer solutions confined in narrower slits. Anticipating a comparison with CG-DFT, in MD for the latter two cases we have simulated slit widths that correspond to a fixed multiple of the polymer’s radius of gyration at infinite dilution, *R*_g,0_^ring/lin^. The organization of ring and linear polymers in terms of their center-of-mass density profiles *h*_CM_(*z*) within slits of width *d*/*R*_g,0_ = 2, 4, and 6 for different average monomer densities ρ is shown in Figure 4. In the narrowest slit that we simulated (*d*/*R*_g,0_ = 2), we find that the polymers at all densities considered tend to be located in the center of the slit in both cases (Figure 4a,d). A somewhat different pattern arises when *d* is increased. In particular, in the case of linear chains, we find two peaks of *h*_CM_(*z*) at the walls and a flat density profile in the middle of the slit for all ρ considered (Figure 4e,f), similar to the results in a very wide slit in Figure 4d. More correlated and oscillatory density profiles are found for rings (Figure 4b,c) that tend to develop a secondary peak in the center of the slit at higher densities, in agreement with previous CG-DFT results.^19^

**Figure 5:**
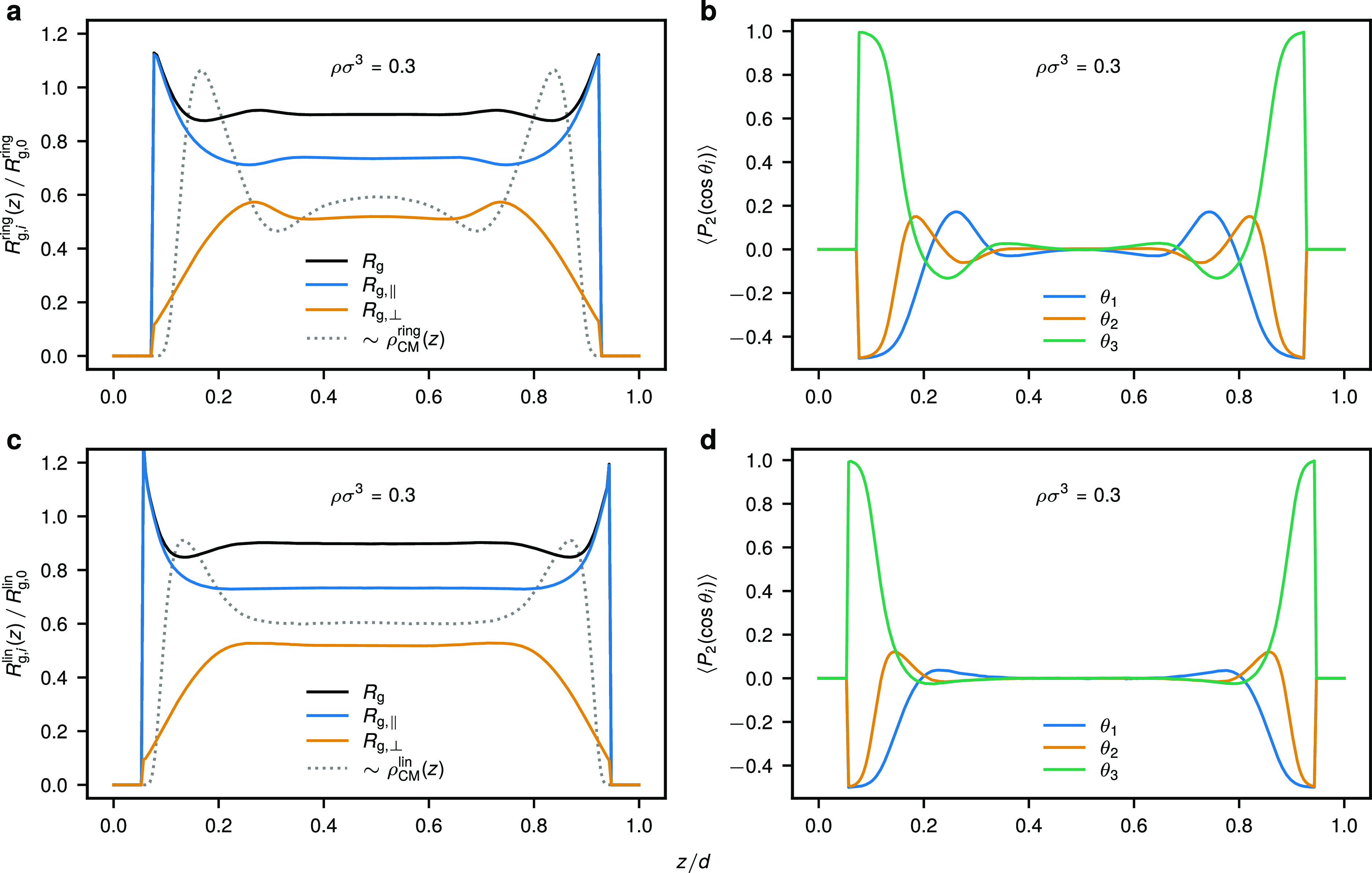
Conformational properties of ring (top row) and linear (bottom row) polymers confined in a slit of width *d* = 6*R*_g,0_^ring^ and 6*R*_g,0_^lin^, respectively, at ρσ^3^ = 0.3. The total *R*_g_, parallel to the wall *R*_g,∥_, and orthogonal to the wall *R*_g,⊥_ radius of gyration of a polymer for (a) rings and (c) linear chains as a function of the distance *z* away from the first wall for ρσ^3^ = 0.3. The dashed curves in (a) and (c) indicate the respective center-of-mass density profiles that have been arbitrarily scaled for clarity. The alignment of the three eigenvalues of a polymer’s gyration tensor with the confining wall quantified by means of the second Legendre polynomial (48) for (b) rings and (d) linear chains as a function of *z* for ρσ^3^ = 0.3. The results presented here were obtained in MD.

Similar to the case of a contact with a single hard wall (Figure 2), we find that more pronounced density correlations in the case of rings are associated with conformational changes in the polymer fluid layers at the wall. We illustrate this in Figure 5 by comparing polymer conformational properties within the slit for both rings and linear chains for *d* = 6*R*_g,0_ at ρσ^3^ = 0.3. For linear chains, we again observe that *R*_g,⊥_ and *R*_g,∥_ monotonously arrive at the bulk value corresponding to the flat region of *h*_CM_(*z*) (Figure 5c). However, for rings we find a nonmonotonic behavior with *R*_g,⊥_ having a maximum close to the region of reduced polymer density (Figure 5a). As in the case of a single wall but more pronounced here, in the latter region the biggest eigenvalues of the ring’s gyration tensor λ_1_ tends to orient in the direction orthogonal to the wall (compare Figure 5 panels b and d).

## 6 Coarse-Grained Viewpoint

From now on, we will adopt a coarse-grained viewpoint on the problem at hand by treating polymers in solution as soft colloids.^29,53,56^ While such a description leads to relatively simple effective pair interactions between the chosen polymer’s coarse-grained degrees of freedom that can allow for systematic analytical treatment, it certainly oversimplifies multibody correlations between the chains, as well as neglects chain deformations that become increasingly important at higher system densities. Nevertheless, as we will show below using a mean-field CG-DFT, even such an approach provides a reasonably good description of ring polymer systems at lower densities when compared to more computationally expensive MD and MR-DFT methods discussed previously.

### 6.1 Polymer Solutions Confined within a Slit

We now consider CG-DFT for polymer solutions confined within a slit of width *d*, that is being in contact with two parallel, hard walls located at *z* = 0 and *d*. In such a geometry, the external potential *V*_ext_(**r**) ≡ *V*_ext_(*z*) in eq 40 becomes
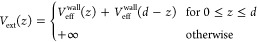
52Such a form of the external potential clearly implies that ρ_CM_(**r**) ≡ ρ_CM_(*z*) (ρ_CM_(*z*) = 0 for *z* < 0 and *z* > *d*) and allows us to recast the original integral eq 44 for 0 < *z* < *d* as follows:^19^
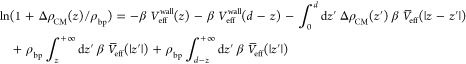
53for 0 ≤ *z* ≤ *d*, where Δρ_CM_(*z*) = ρ_CM_(*z*) – ρ_bp_ and β *V̅*_eff_(|*z* – *z*′|) = ∫_–∞_^+∞^ d*x*′ ∫_–∞_^+∞^ d*y*′ β *V*_eff_(|**r** – **r**′|). More details on the derivation of eq 53 and on CG-DFT used here are available in ref (19). The integral eq 53 is solved iteratively until the tolerance criterion set to 10^–6^ between two consecutive iterations is satisfied.

**Figure 6:**
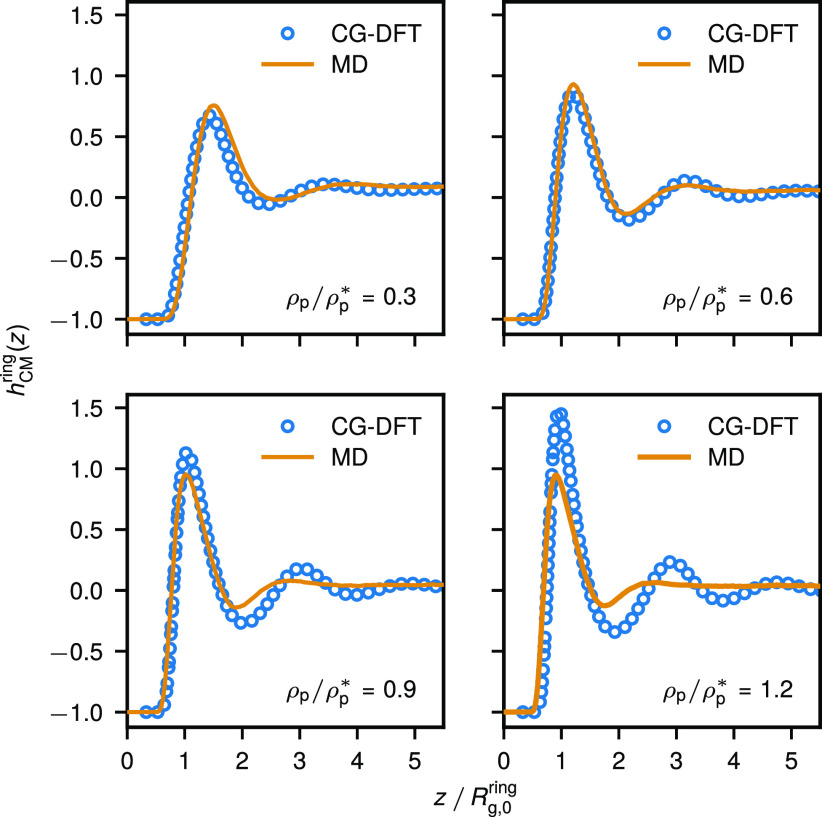
Center-of-mass density profiles for ring polymers in a broad slit of width *d* ≈ 16.3*R*_g,0_^ring^ (effectively resembles a contact with a single hard wall) from monomer-resolved MD (solid lines) and CG-DFT (open circles) for different mean polymer densities ρ_p_ in the slit.

**Figure 7:**
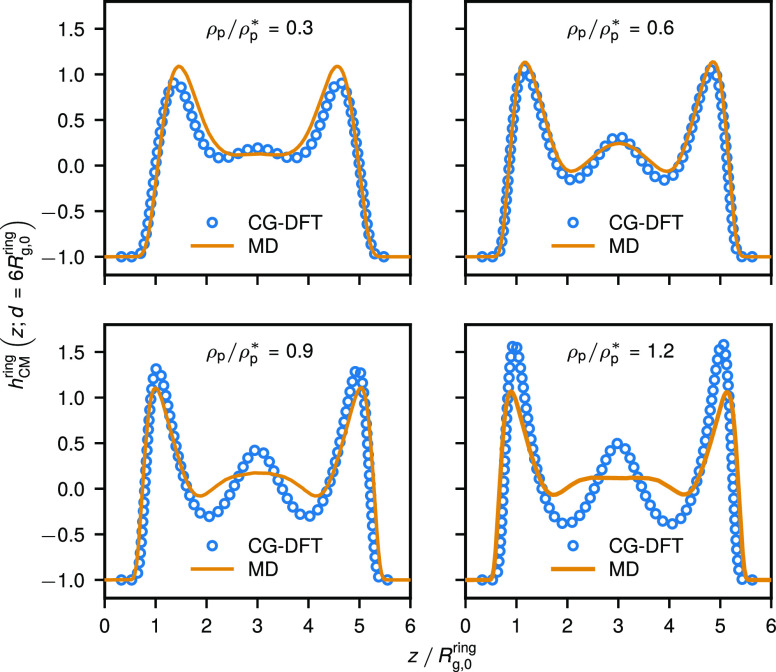
Center-of-mass density profiles for ring polymers confined in a slit of width *d* = 6*R*_g,0_^ring^ from monomer-resolved MD (solid lines) and CG-DFT (open circles) for different mean polymer densities ρ_p_ in the slit.

Figure 6 shows the resulting CG-DFT center-of-mass density profiles *h*_CM_(*z*) = ρ_CM_(*z*)/ρ_p_ – 1 for ring polymers in comparison to the MD results in a broad slit of width *d* = 50σ ≈ 16.3 *R*_g,0_^ring^ at various polymer densities. In such a wide slit, two hard walls are essentially independent from each other. To compare MD and CG-DFT results, we first matched the slit width in CG-DFT (*d*_CG–DFT_ ≈ *d*_MD_ – 2σ due to differences in the monomer–wall interaction potentials) and then determined the bulk polymer density ρ_bp_ that corresponds to the same average polymer density ρ_p_ = *d*^–1^ ∫_0_^*d*^ d*z* ρ_CM_(*z*) as in MD. Figure 6 contains density profiles from CG-DFT and MD at ρ_p_/ρ_p_^*^ = 0.3, 0.6, 0.9, and 1.2 that correspond to the MD monomer densities ρσ^3^ = 0.1, 0.2, 0.3, and 0.4, respectively. We find very good agreement between the two approaches at the two lower concentrations in the dilute regime. A similar comparison is found for narrower slits (see Figure 7 for *d* = 6 *R*_g,0_^ring^ and Figure S3 for *d* = 4 *R*_g,0_^ring^), confirming the validity of a simple mean-field theory (40) based on the infinite-dilution interaction potentials below the semidilute regime. As already shown in Figure 4 and in ref (19), even under such relatively dilute conditions rings feature a higher propensity to structure at the confining walls as compared to equally sized linear chains. Clearly, as we consider ring polymer solutions that approach the semidilute regime (ρ_p_ ≳ ρ_p_^*^), CG-DFT fails by overestimating the structuring effect and the oscillatory character of density profiles. In addition, CG-DFT does not capture pronounced chain deformations near the walls that ultimately lead to tighter packing and stronger layering of polymers at the surface in MD and MR-DFT. Nevertheless, a quite good accuracy of CG-DFT at densities below the overlap concentration ρ_p_^*^ gives confidence in employing such approach in the estimation of ring-polymer-induced depletion interactions between two walls or two colloidal particles. The latter case is of particular interest, as for large colloid–polymer size ratios the monomer-based simulations are not feasible computationally.

### 6.2 Surface Tension of the Interface

DFT is particularly suited for computing thermodynamic quantities that can be expressed in terms of grand potential differences, such as the surface tension of the wall–liquid interface or the fluid-induced depletion potential between the walls, as the grand potential Ω is readily available from equilibrium density profiles. Therefore, in what follows we compare the results from CG-DFT and MR-DFT at different bulk polymer densities ρ_bp_ for both ring and linear polymer solutions. In this section, we will focus on the surface tension of the interface between a hard wall and a polymer fluid, γ, whereas in the following one we will return to the determination of depletion potentials.

Let us consider a hard wall in contact with a fluid at one side of it, so the total system volume is *V* = *A* × *L* with *A* as the area of the interface and *L* as the system length in the direction orthogonal to the wall. Then, γ can be obtained as the difference between the grand potential of the system per unit area with and without the wall:^39^
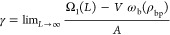
54where Ω_1_(*L*) is the grand potential of the fluid in contact with a hard wall, and ω_b_ is the grand potential density of the bulk fluid, ω_b_ ≡ Ω_b_/*V* (note that ω_b_ = −*p* with *p* being the pressure of the bulk fluid). In the case of CG-DFT, we have computed the equilibrium density profiles using an integral equation similar to eq 53 but without the contribution of the second wall located at *z* = *d* (more specifically, without the terms −β *V*_eff_^wall^(*d* – *z*) + ρ_bp_∫_*d*–*z*_^+∞^ d*z*′ β *V̅*_eff_(|*z*′|) on the right-hand side of eq 53) and used them to evaluate the corresponding grand potential difference (eq 54). A more elaborate discussion on the determination of γ for the given mean-field CG-DFT is available in ref (19).

**Figure 8:**
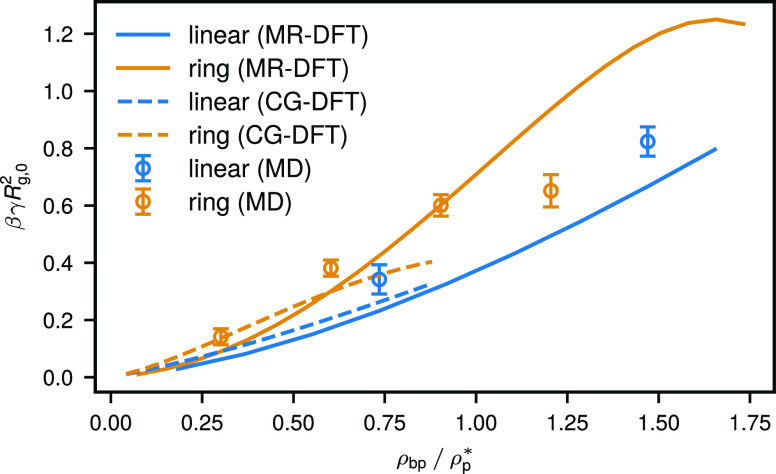
Surface tension for ring and linear solutions in contact with a hard repulsive wall as extracted from MR-DFT (solid lines) and CG-DFT (dashed lines) for different bulk polymer densities ρ_bp_ and MD (open circles) at different average polymer densities ρ_p_.

The surface tension of the interface for ring and linear polymers both from CG-DFT and MR-DFT is shown in Figure 8 and compared to earlier MD results. We find good agreement between all methods for ρ_p_ ≲ ρ_p_^*^. Interestingly, when the surface tension for the two architectures considered is expressed in terms of the volume fraction of the polymer component, the trend found in Figure 3 is reversed, and the rings appear as stronger depletants.

### 6.3 Polymer-Induced Depletion Potentials between the Walls

We finally consider the polymer-induced depletion potentials between the two confining walls. The depletion potential *V*_dep_(*d*) in this case is caused by the reduced entropy of the polymer fluid confined between two impenetrable walls as compared to the bulk. Let us consider two hard walls immersed in a polymer solution at a distance *d* in a system of total volume *V* = *A* × *L*, where *A* is the cross-sectional area of the box and *L* is the box length. The depletion potential *V*_dep_(*d*) is defined as the difference between the grand potential Ω(*d*) of the system for a given separation *d* and the value of the grand potential when the walls are far away from each other, Ω(*d* → ∞), given that the system volume *V* is constant:^63^
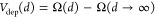
55For a given value of *d*, we can write Ω(*d*) = Ω_2_(*d*) + Ω_rest_(*d*), where Ω_2_(*d*) in the grand potential contribution arising from the fluid in the region within the two walls and Ω_rest_(*d*) is the grand potential of the fluid outside. The latter term contains the cost of forming two interfaces, 2γ*A*, and the grand potential of the bulk fluid in the remaining volume, ω_b_(ρ_bp_) *A*(*L* – *d*), i.e. Ω_rest_(*d*) = 2γ*A* + ω_b_(ρ_bp_) *A*(*L* – *d*). Here, ω_b_(ρ_bp_) ≡ Ω_b_/*V* is the grand potential density, which equals ω_b_(ρ_bp_) ≡ −*p*, with *p* being the pressure of the bulk fluid. In the case of the mean-field CG-DFT employed here, we evaluated the equilibrium density profiles that satisfy eq 53 and used them to compute the associated grand potential difference as defined above. A more detailed discussion on the determination of *V*_dep_(*d*) for the given mean-field CG-DFT is available in ref (19).

**Figure 9:**
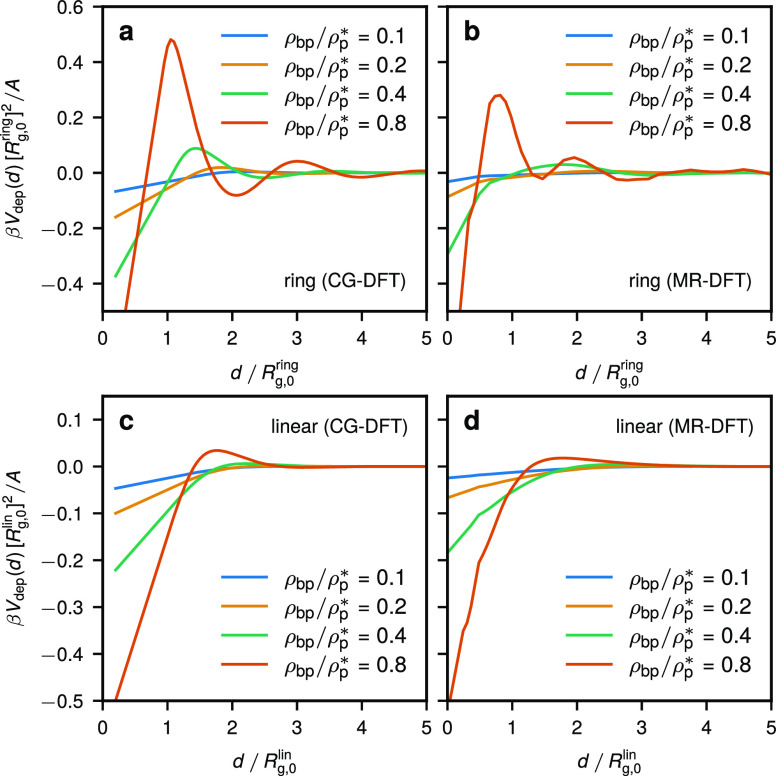
Polymer-induced depletion potentials between two hard walls. Top row: *V*_dep_(*d*) for ring polymers obtained from CG-DFT (a) and MR-DFT (b) at different bulk polymer densities ρ_bp_. Bottom row: *V*_dep_(*d*) for linear polymers as obtained from CG-DFT (c) and MR-DFT (d) at different bulk polymer densities ρ_bp_.

The resulting depletion potentials per unit area from CG-DFT and MR-DFT are shown in Figure 9. Both CG-DFT and MR-DFT predict a considerably different form of the depletion potential for rings as compared to linear chains. In particular, at the highest density considered here, ρ_bp_/ρ_p_^*^ = 0.8, we find an oscillatory structure of the depletion potential in the case of rings, a feature not seen for the linear counterparts, as well as a much higher peak of *V*_dep_(*d*). Evidently, such differences arise from a higher propensity of rings to structure in confinement, as already seen from the equilibrium density profiles in Figure 2 when the two architectures are compared. While MR-DFT qualitatively confirms the results from CG-DFT for rings and the oscillatory structure of the depletion potential, certain differences are found. More specifically, for ρ_bp_/ρ_p_^*^ = 0.8, we find a lower amplitude of he repulsive part of *V*_dep_(*d*), which is about 0.3*k*_B_*T* for MR-DFT and 0.5*k*_B_*T* for CG-DFT, as well as a somewhat different location of its peaks. Such differences likely stem from the breakdown of the mean-field CG-DFT at densities approaching the overlap concentration ρ_p_^*^, when non-negligible polymer deformations and interchain correlations change the character of the effective inter-ring interaction. Finally, at comparable volume fractions of the polymer component we find a lower depth of the depletion potential lim_*d*→0_*V*_dep_(*d*) = −2γ for rings.

## 7 Discussion and Conclusions

In summary, we have considered confined ring and linear polymer solutions, with the emphasis put on the case of ring architecture, using a series of multiscale modeling approaches. First, we have developed MR-DFT for ring polymer chains of finite length *N*, as given by the equilibrium segment density (eq 26) with the corresponding propagators (eqs 27 and 28). MR-DFT yields very good agreement with MD simulations both in terms of the monomer density profiles (Figure 1) as well as the surface tension at the solution–wall interface (Figure 3). Some discrepancy in the surface tension results is observed at higher densities, which might be attributed to the approximations employed in the EOS. We then compared mean-field CG-DFT, which models polymer coils as soft colloids, developed in ref (19), to explicit MD results. CG-DFT provides a good description for the structure of ring polymer solutions within slits of variable width up to ρ_p_/ρ_p_^*^ ≲ 0.6–0.8 (Figure 6), despite polymer deformations in the vicinity of the walls found in MD (Figure 2). A rather small impact of the latter on the final density profiles from CG-DFT (eq 40) is likely due to the fact that the profiles at close polymer–wall separations mainly depend on the effective polymer–wall potential, which implicitly includes the conformational rearrangements.^19^ In addition, the surface tension obtained in CG-DFT is, within the range of applicable densities, in good agreement with those obtained in the MR-DFT and MD (Figure 8). The rings feature a more pronounced tendency to structure at the walls as compared to linear chains, as shown here with MD and CG-DFT results. The latter feature strongly affects the form of the depletion potential between two walls immersed in a ring polymer solution that we computed using the monomer-resolved and coarse-grained versions of DFT (Figure 9). The depletion potentials from CG-DFT compare quite well to the ones from the MR-DFT. Finally, we clarified the comparison between the values of *V*_dep_(*d*) at *d* = 0, i.e., the strength of the depletion attraction between the walls, for the linear and ring polymer architectures. At a fixed monomer density ρ, we find that linear chains appear as stronger depletants than do rings due to their enhanced surface tension at the interface (Figure 3). In contrast, when the results are compared at a fixed polymer concentration in terms of the overlap one, i.e., at comparable volume fractions of the polymer component, the rings turn out to be stronger depletants than do the linear chains (Figure 8), in full agreement with previous results.^19^ The latter difference can be important in the interpretation of potential experimental findings for the polymer-induced depletion effects in colloidal mixtures. In practical terms, our results suggest that colloidal gels with ring polymer additives should feature enhanced values of the storage modulus when compared to the ones with linear polymer additives at a similar volume fraction of polymers. Future work should focus on the generalization of these considerations to mixtures of ring polymers and spherical colloids with an arbitrary size ratio between the two components to gain a better understanding of the stability and elasticity of such systems.

## Supporting Information Available

Organization of ring and linear polymer chains in contact with a hard repulsive wall at the average monomer densities of ρσ^3^ = 0.1 and 0.6; center-of-mass density profiles (PDF)

## References

[ref1] Halverson J. D.; Lee W. B.; Grest G. S.; Grosberg A. Y.; Kremer K. Molecular dynamics simulation study of nonconcatenated ring polymers in a melt. I. Statics. J. Chem. Phys. 2011, 134, 204904 10.1063/1.3587137. 21639474

[ref2] Rosa A.; Everaers R. Ring Polymers in the Melt State: The Physics of Crumpling. Phys. Rev. Lett. 2014, 112, 118302 10.1103/PhysRevLett.112.118302. 24702424

[ref3] Pachong S. M.; Chubak I.; Kremer K.; Smrek J. Melts of nonconcatenated rings in spherical confinement. J. Chem. Phys. 2020, 153, 064903 10.1063/5.0013929. 35287461

[ref4] Halverson J. D.; Lee W. B.; Grest G. S.; Grosberg A. Y.; Kremer K. Molecular dynamics simulation study of nonconcatenated ring polymers in a melt. II Dynamics. J. Chem. Phys. 2011, 134, 204905 10.1063/1.3587138. 21639475

[ref5] Smrek J.; Grosberg A. Y. Understanding the dynamics of rings in the melt in terms of the annealed tree model. J. Phys.: Condens. Matter 2015, 27, 064117 10.1088/0953-8984/27/6/064117. 25563563

[ref6] Kapnistos M.; Lang M.; Vlassopoulos D.; Pyckhout-Hintzen W.; Richter D.; Cho D.; Chang T.; Rubinstein M. Unexpected Power-Law Stress Relaxation of Entangled Ring Polymers. Nat. Mater. 2008, 7, 997–1002. 10.1038/nmat2292. 18953345 PMC4819970

[ref7] Grosberg A. Y. Annealed lattice animal model and Flory theory for the melt of non-concatenated rings: towards the physics of crumpling. Soft Matter 2014, 10, 560–565. 10.1039/C3SM52805G. 24652534

[ref8] Ge T.; Panyukov S.; Rubinstein M. Self-Similar Conformations and Dynamics in Entangled Melts and Solutions of Nonconcatenated Ring Polymers. Macromolecules 2016, 49, 708–722. 10.1021/acs.macromol.5b02319. 27057066 PMC4819263

[ref9] Lang M. Ring Conformations in Bidisperse Blends of Ring Polymers. Macromolecules 2013, 46, 1158–1166. 10.1021/ma301359b.

[ref10] Smrek J.; Grosberg A. Y. Minimal surfaces on unconcatenated polymer rings in melt. ACS Macro Lett. 2016, 5, 750–754. 10.1021/acsmacrolett.6b00289. 35614671

[ref11] Michieletto D.; Marenduzzo D.; Orlandini E.; Alexander G. P.; Turner M. S. Threading Dynamics of Ring Polymers in a Gel. ACS Macro Lett. 2014, 3, 255–259. 10.1021/mz500060c. 35590516

[ref12] Lee E.; Kim S.; Jung Y. Slowing Down of Ring Polymer Diffusion Caused by Inter-Ring Threading. Macromol. Rapid Commun. 2015, 36, 1115–1121. 10.1002/marc.201400713. 25881785

[ref13] Michieletto D.; Turner M. S. A topologically driven glass in ring polymers. Proc. Natl. Acad. Sci. U. S. A. 2016, 113, 5195–5200. 10.1073/pnas.1520665113. 27118847 PMC4868430

[ref14] O’Connor T. C.; Ge T.; Rubinstein M.; Grest G. S. Topological Linking Drives Anomalous Thickening of Ring Polymers in Weak Extensional Flows. Phys. Rev. Lett. 2020, 124, 027801 10.1103/PhysRevLett.124.027801. 32004030 PMC7190399

[ref15] Smrek J.; Chubak I.; Likos C. N.; Kremer K. Active topological glass. Nat. Commun. 2020, 11, 26 10.1038/s41467-019-13696-z. 31911582 PMC6946665

[ref16] Chubak I.; Likos C. N.; Kremer K.; Smrek J. Emergence of active topological glass through directed chain dynamics and nonequilibrium phase segregation. Phys. Rev. Research 2020, 2, 043249 10.1103/PhysRevResearch.2.043249.

[ref17] Narros A.; Moreno A. J.; Likos C. N. Architecture-induced size asymmetry and effective interactions of ring polymers: simulation and theory. Macromolecules 2013, 46, 9437–9445. 10.1021/ma4016483. 24347686 PMC3859368

[ref18] Narros A.; Moreno A. J.; Likos C. N. Effects of Knots on Ring Polymers in Solvents of Varying Quality. Macromolecules 2013, 46, 3654–3668. 10.1021/ma400308x. 23729865 PMC3667624

[ref19] Chubak I.; Locatelli E.; Likos C. N. Ring polymers are much stronger depleting agents than linear ones. Mol. Phys. 2018, 116, 2911–2926. 10.1080/00268976.2018.1503744.

[ref20] Ahmadian Dehaghani Z.; Chubak I.; Likos C. N.; Ejtehadi M. R. Effects of topological constraints on linked ring polymers in solvents of varying quality. Soft Matter 2020, 16, 3029–3038. 10.1039/C9SM02374G. 32129365

[ref21] Weiss L. B.; Likos C. N.; Nikoubashman A. Spatial Demixing of Ring and Chain Polymers in Pressure-Driven Flow. Macromolecules 2019, 52, 7858–7869. 10.1021/acs.macromol.9b01629.

[ref22] Liebetreu M.; Likos C. N. Hydrodynamic inflation of ring polymers under shear. Comms. Mater. 2020, 1, 4 10.1038/s43246-019-0006-5.

[ref23] Tu M. Q.; Lee M.; Robertson-Anderson R. M.; Schroeder C. M. Direct Observation of Ring Polymer Dynamics in the Flow-Gradient Plane of Shear Flow. Macromolecules 2020, 53, 9406–9419. 10.1021/acs.macromol.0c01362.

[ref24] Grosberg A. Y. Critical Exponents for Random Knots. Phys. Rev. Lett. 2000, 85, 3858–3861. 10.1103/PhysRevLett.85.3858. 11041945

[ref25] Deutsch J. M. Equilibrium size of large ring molecules. Phys. Rev. E: Stat. Phys., Plasmas, Fluids, Relat. Interdiscip. Top. 1999, 59, R2539–R2541. 10.1103/PhysRevE.59.R2539.

[ref26] Bohn M.; Heermann D. W. Topological interactions between ring polymers: Implications for chromatin loops. J. Chem. Phys. 2010, 132, 044904 10.1063/1.3302812. 20113063

[ref27] Narros A.; Moreno A. J.; Likos C. N. Influence of topology on effective potentials: coarse-graining ring polymers. Soft Matter 2010, 6, 2435–2441. 10.1039/c001523g.

[ref28] Chubak I.; Likos C. N.; Smrek J. Topological and threading effects in polydisperse ring polymer solutions. Mol. Phys. 2021, 0, e1883140 10.1080/00268976.2021.1883140.

[ref29] Likos C. N. Effective interactions in soft condensed matter physics. Phys. Rep. 2001, 348, 267–439. 10.1016/S0370-1573(00)00141-1.

[ref30] Patel N.; Egorov S. A. Interactions between colloidal particles in polymer solutions: A density functional theory study. J. Chem. Phys. 2004, 121, 4987–4997. 10.1063/1.1778671. 15332935

[ref31] Patel N.; Egorov S. A. Dispersing Nanotubes with Surfactants: A Microscopic Statistical Mechanical Analysis. J. Am. Chem. Soc. 2005, 127, 14124–14125. 10.1021/ja0530570. 16218573

[ref32] Patel N.; Egorov S. A. Interactions between nanocolloidal particles in polymer solutions: Effect of attractive interactions. J. Chem. Phys. 2005, 123, 144916 10.1063/1.2049275. 16238433

[ref33] Egorov S. A.; Milchev A.; Binder K. Anomalous Fluctuations of Nematic Order in Solutions of Semiflexible Polymers. Phys. Rev. Lett. 2016, 116, 187801 10.1103/PhysRevLett.116.187801. 27203343

[ref34] Jiang J.; Xu X.; Cao D. Density functional theory for inhomogeneous ring polymeric fluids. Phys. Rev. E 2012, 86, 041805 10.1103/PhysRevE.86.041805. 23214608

[ref35] Honnell K. G.; Hall C. K. Theory and simulation of hard?chain mixtures: Equations of state, mixing properties, and density profiles near hard walls. J. Chem. Phys. 1991, 95, 4481–4501. 10.1063/1.461772.

[ref36] Carnahan N. F.; Starling K. E. Equation of State for Nonattracting Rigid Spheres. J. Chem. Phys. 1969, 51, 635–636. 10.1063/1.1672048.

[ref37] Tildesley D.; Streett W. An equation of state for hard dumbell fluids. Mol. Phys. 1980, 41, 85–94. 10.1080/00268978000102591.

[ref38] Yethiraj A.; Woodward C. E. Monte Carlo density functional theory of nonuniform polymer melts. J. Chem. Phys. 1995, 102, 5499–5505. 10.1063/1.469279.

[ref39] Hansen J.-P.; McDonald I. R. Theory of Simple Liquids, 4th ed.; Elsevier: Amsterdam, 2013.

[ref40] Evans R. The nature of the liquid-vapour interface and other topics in the statistical mechanics of non-uniform, classical fluids. Adv. Phys. 1979, 28, 143–200. 10.1080/00018737900101365.

[ref41] Evans R. In Fundamentals of Inhomogeneous Fluids; Henderson D., Ed.; Dekker: New York, 1992; Chapter 3, pp 85.

[ref42] Milchev A.; Egorov S. A.; Binder K. Absorption/expulsion of oligomers and linear macromolecules in a polymer brush. J. Chem. Phys. 2010, 132, 184905 10.1063/1.3414996.

[ref43] Woodward C. E. A density functional theory for polymers: Application to hard chain – hard sphere mixtures in slitlike pores. J. Chem. Phys. 1991, 94, 3183–3191. 10.1063/1.459787.

[ref44] Egorov S. A. Interactions between polymer brushes in solvents of variable quality: A density functional theory study. J. Chem. Phys. 2008, 129, 064901 10.1063/1.2968545. 18715103

[ref45] LoVerso F.; Egorov S. A.; Binder K. Interaction Between Polymer Brush-Coated Spherical Nanoparticles: Effect of Solvent Quality. Macromolecules 2012, 45, 8892–8902. 10.1021/ma301651z.

[ref46] Roth R. Fundamental measure theory for hard-sphere mixtures: a review. J. Phys.: Condens. Matter 2010, 22, 063102 10.1088/0953-8984/22/6/063102. 21389360

[ref47] Turesson M.; Forsman J.; Åkesson T. Simulations and density functional calculations of surface forces in the presence of semiflexible polymers. Phys. Rev. E 2007, 76, 021801 10.1103/PhysRevE.76.021801. 17930055

[ref48] Woodward C. E.; Forsman J. Density functional theory for flexible and semiflexible polymers of infinite length. Phys. Rev. E 2006, 74, 010801 10.1103/PhysRevE.74.010801. 16907051

[ref49] Grest G. S.; Kremer K. Molecular dynamics simulation for polymers in the presence of a heat bath. Phys. Rev. A: At., Mol., Opt. Phys. 1986, 33, 3628–3631. 10.1103/PhysRevA.33.3628. 9897103

[ref50] Anderson J. A.; Glaser J.; Glotzer S. C. HOOMD-blue: A Python package for high-performance molecular dynamics and hard particle Monte Carlo simulations. Comput. Mater. Sci. 2020, 173, 109363 10.1016/j.commatsci.2019.109363.

[ref51] Howard M. P.; Anderson J. A.; Nikoubashman A.; Glotzer S. C.; Panagiotopoulos A. Z. Efficient neighbor list calculation for molecular simulation of colloidal systems using graphics processing units. Comput. Phys. Commun. 2016, 203, 45–52. 10.1016/j.cpc.2016.02.003.

[ref52] Narros A.; Moreno A. J.; Likos C. N. Effective interactions of knotted ring polymers. Biochem. Soc. Trans. 2013, 41, 630–634. 10.1042/BST20120286. 23514167

[ref53] Louis A. A.; Bolhuis P. G.; Hansen J. P.; Meijer E. J. Can polymer coils be modeled as “soft colloids”?. Phys. Rev. Lett. 2000, 85, 2522–2525. 10.1103/PhysRevLett.85.2522. 10978097

[ref54] Lang A.; Likos C. N.; Watzlawek M.; Löwen H. Fluid and solid phases of the Gaussian core model. J. Phys.: Condens. Matter 2000, 12, 5087 10.1088/0953-8984/12/24/302.

[ref55] Likos C.; Lang A.; Watzlawek M.; Löwen H. Criterion for determining clustering versus reentrant melting behavior for bounded interaction potentials. Phys. Rev. E: Stat. Phys., Plasmas, Fluids, Relat. Interdiscip. Top. 2001, 63, 031206 10.1103/PhysRevE.63.031206. 11308641

[ref56] Louis A. A.; Bolhuis P. G.; Hansen J. P. Mean-field fluid behavior of the Gaussian core model. Phys. Rev. E: Stat. Phys., Plasmas, Fluids, Relat. Interdiscip. Top. 2000, 62, 7961–7972. 10.1103/PhysRevE.62.7961. 11138080

[ref57] Archer A. J.; Evans R. Binary Gaussian core model: Fluid-fluid phase separation and interfacial properties. Phys. Rev. E: Stat. Phys., Plasmas, Fluids, Relat. Interdiscip. Top. 2001, 64, 041501 10.1103/PhysRevE.64.041501. 11690027

[ref58] Bolhuis P. G.; Louis A. A. How To Derive and Parameterize Effective Potentials in Colloid?Polymer Mixtures. Macromolecules 2002, 35, 1860–1869. 10.1021/ma010888r.

[ref59] Egorov S. A.; Milchev A.; Virnau P.; Binder K. Semiflexible polymers under good solvent conditions interacting with repulsive walls. J. Chem. Phys. 2016, 144, 174902 10.1063/1.4947254. 27155651

[ref60] Irving J. H.; Kirkwood J. G. The Statistical Mechanical Theory of Transport Processes. IV. The Equations of Hydrodynamics. J. Chem. Phys. 1950, 18, 817–829. 10.1063/1.1747782.

[ref61] Varnik F.; Baschnagel J.; Binder K. Molecular dynamics results on the pressure tensor of polymer films. J. Chem. Phys. 2000, 113, 4444–4453. 10.1063/1.1288390.

[ref62] Milchev A. Effects of polymer stiffness on surface tension and pressure in confinement. J. Chem. Phys. 2015, 143, 064701 10.1063/1.4927559. 26277150

[ref63] Roth R.; Evans R.; Dietrich S. Depletion potential in hard-sphere mixtures: Theory and applications. Phys. Rev. E: Stat. Phys., Plasmas, Fluids, Relat. Interdiscip. Top. 2000, 62, 5360–5377. 10.1103/PhysRevE.62.5360. 11089098

