# Supplementary material for: Multiscale Approaches for Confined Ring Polymer Solutions
Source: J Phys Chem B. 2021 May 3;125(18):4910–23. doi: 10.1021/acs.jpcb.1c01953 (PMC8279562; doi:10.1021/acs.jpcb.1c01953)
Supplement: Supplementary file 1 — jp1c01953_si_001.pdf [file jp1c01953_si_001.pdf]

Supporting information for: Multiscale approaches for confined ring polymer solutions

Iurii Chubak,[*,†,‡] Christos N. Likos,[*,†] and Sergei A. Egorov[*,¶]

†*Faculty of Physics, University of Vienna, Boltzmanngasse 5, A-1090 Vienna, Austria*

‡*Sorbonne Université CNRS, Physico-Chimie des électrolytes et Nanosystèmes Interfaciaux, F-75005 Paris, France*

¶*Department of Chemistry, University of Virginia, Charlottesville, Virginia 22901, USA*

E-mail: iurii.chubak@sorbonne-universite.fr; christos.likos@univie.ac.at; sae6z@virginia.edu

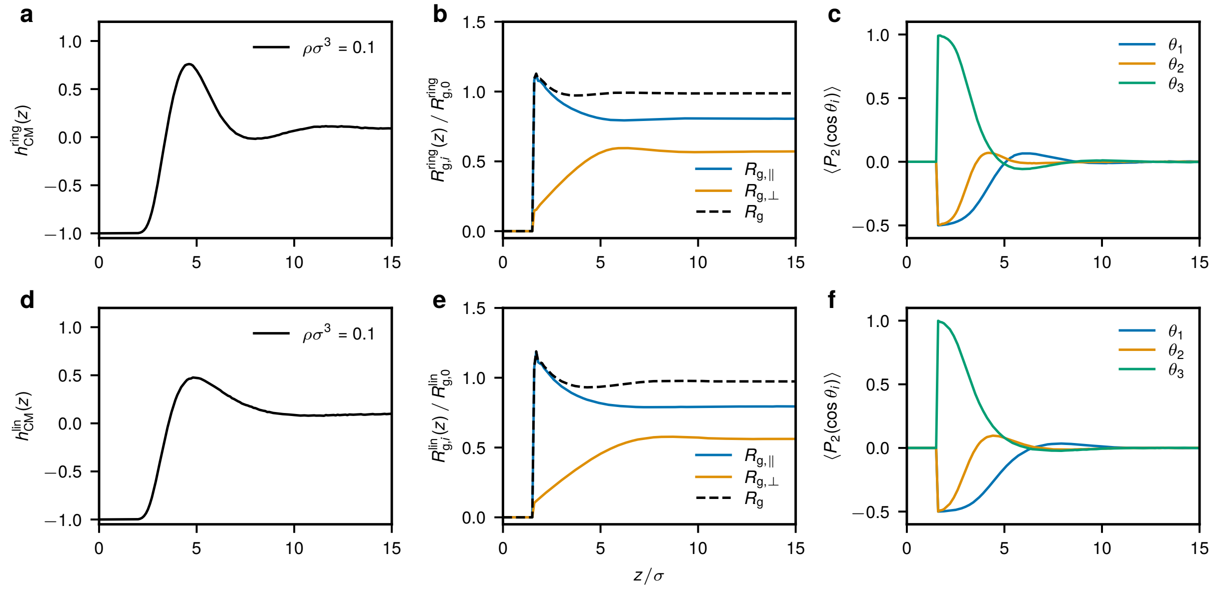

Figure S1: Organization of ring (top row) and linear (bottom row) polymer chains in contact with a hard repulsive wall at the average monomer density $\rho\sigma^3 = 0.1$. Center-of-mass density profiles for (**a**) ring and (**d**) linear polymers. The total $R_{\rm g}$, parallel to the wall $R_{{\rm g},\parallel}$, and orthogonal to the wall $R_{{\rm g},\perp}$ radius of gyration of a polymer for (**b**) rings and (**e**) linear chains as a function of the distance z away from the wall. The alignment of the three eigenvalues of a polymer's gyration tensor with the confining wall quantified by means of the second Legendre polynomial (48) for (**c**) rings and (**f**) linear chains as a function of z. The results presented here correspond to polymer solutions confined in a broad slit of width $d = 50\sigma$. The results presented here were obtained in MD.

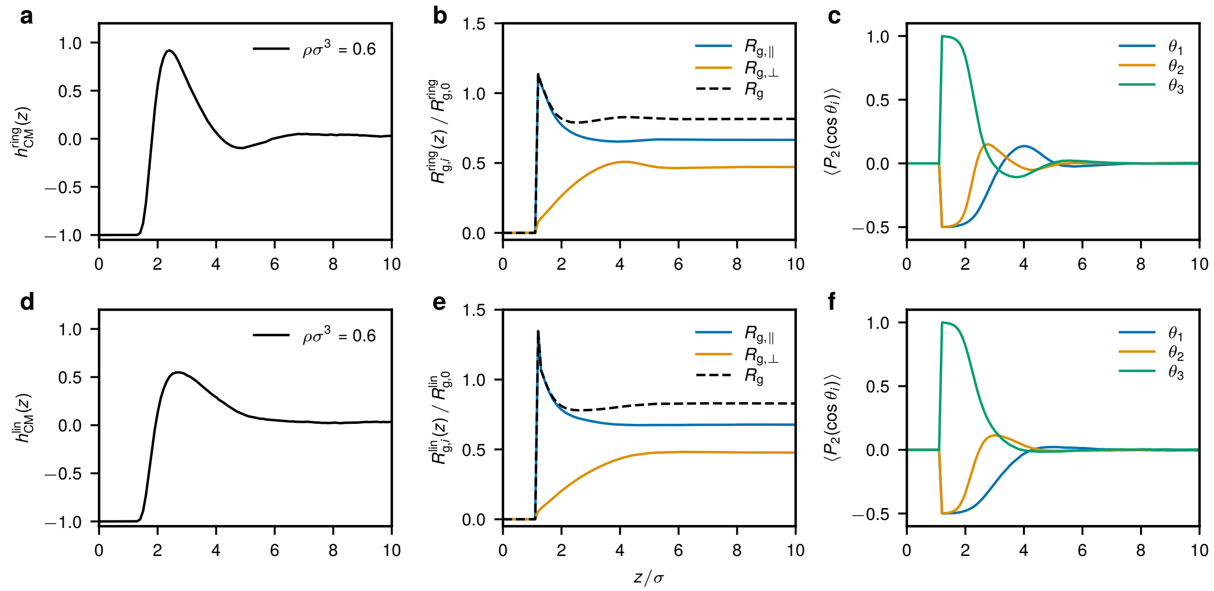

Figure S2: Organization of ring (top row) and linear (bottom row) polymer chains in contact with a hard repulsive wall at the average monomer density $\rho\sigma^3 = 0.6$. Center-of-mass density profiles for (**a**) ring and (**d**) linear polymers. The total $R_{\rm g}$, parallel to the wall $R_{{\rm g},\parallel}$, and orthogonal to the wall $R_{{\rm g},\perp}$ radius of gyration of a polymer for (**b**) rings and (**e**) linear chains as a function of the distance z away from the wall. The alignment of the three eigenvalues of a polymer's gyration tensor with the confining wall quantified by means of the second Legendre polynomial (48) for (**c**) rings and (**f**) linear chains as a function of z. The results presented here correspond to polymer solutions confined in a broad slit of width $d = 50\sigma$. The results presented here were obtained in MD.

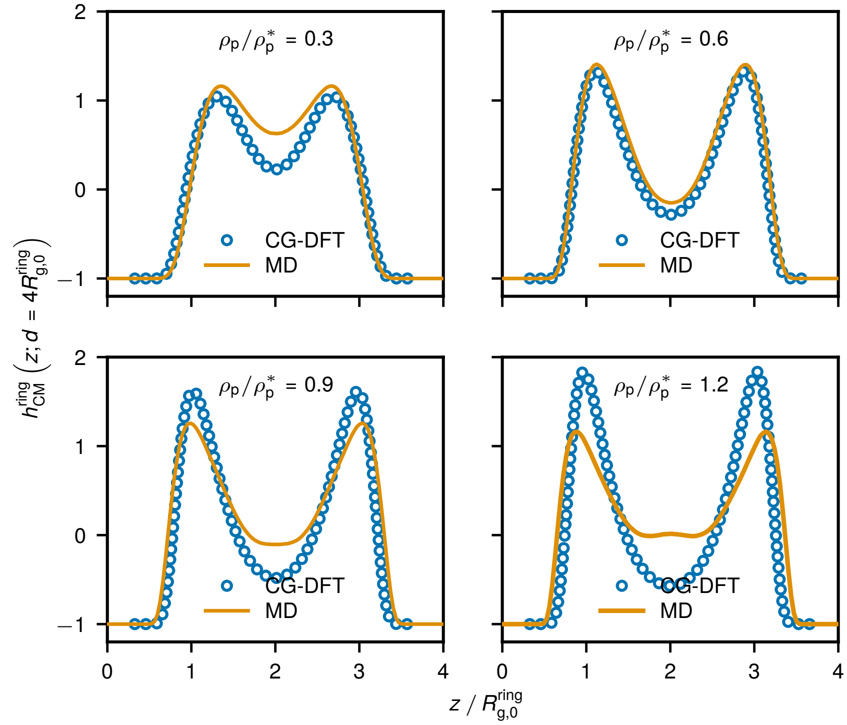

Figure S3: Center-of-mass density profiles for ring polymers confined in a slit of width $d = 4R_{g,0}^{\text{ring}}$ and CG-DFT (open circles) for different mean polymer densities ρ_p in the slit.
